# Oximes: Novel Therapeutics with Anticancer and Anti-Inflammatory Potential

**DOI:** 10.3390/biom11060777

**Published:** 2021-05-22

**Authors:** Igor A. Schepetkin, Mark B. Plotnikov, Andrei I. Khlebnikov, Tatiana M. Plotnikova, Mark T. Quinn

**Affiliations:** 1 Department of Microbiology and Cell Biology, Montana State University, Bozeman, MT 59717, USA; igor@montana.edu; 2 Goldberg Research Institute of Pharmacology and Regenerative Medicine, Tomsk National Research Medical Center, Russian Academy of Sciences, 634028 Tomsk, Russia; mbp2001@mail.ru; 3 Kizhner Research Center, National Research Tomsk Polytechnic University, 634050 Tomsk, Russia; aikhl@chem.org.ru; 4 Scientific Research Institute of Biological Medicine, Altai State University, 656049 Barnaul, Russia; 5 Department of Pharmacology, Siberian State Medical University, 634050 Tomsk, Russia; plot160@mail.ru

**Keywords:** oxime, kinase inhibitor, indirubin, nitric oxide, molecular modeling, inflammation, cancer

## Abstract

Oximes have been studied for decades because of their significant roles as acetylcholinesterase reactivators. Over the last twenty years, a large number of oximes have been reported with useful pharmaceutical properties, including compounds with antibacterial, anticancer, anti-arthritis, and anti-stroke activities. Many oximes are kinase inhibitors and have been shown to inhibit over 40 different kinases, including AMP-activated protein kinase (AMPK), phosphatidylinositol 3-kinase (PI3K), cyclin-dependent kinase (CDK), serine/threonine kinases glycogen synthase kinase 3 α/β (GSK-3α/β), Aurora A, B-Raf, Chk1, death-associated protein-kinase-related 2 (DRAK2), phosphorylase kinase (PhK), serum and glucocorticoid-regulated kinase (SGK), Janus tyrosine kinase (JAK), and multiple receptor and non-receptor tyrosine kinases. Some oximes are inhibitors of lipoxygenase 5, human neutrophil elastase, and proteinase 3. The oxime group contains two H-bond acceptors (nitrogen and oxygen atoms) and one H-bond donor (OH group), versus only one H-bond acceptor present in carbonyl groups. This feature, together with the high polarity of oxime groups, may lead to a significantly different mode of interaction with receptor binding sites compared to corresponding carbonyl compounds, despite small changes in the total size and shape of the compound. In addition, oximes can generate nitric oxide. This review is focused on oximes as kinase inhibitors with anticancer and anti-inflammatory activities. Oximes with non-kinase targets or mechanisms of anti-inflammatory activity are also discussed.

## 1. Introduction

Oxime compounds have been investigated for decades because of their significant roles as acetylcholinesterase reactivators and their use as therapeutics for a number of diseases [1,2,3]. Metabolites of various oximes have also been identified in plants as intermediates in biosynthesis and can facilitate a range of processes important for plant growth and development (for review [4]). Since amidoximes were found to be synthetic antimicrobial agents [5], oximes with different scaffolds have been developed for the treatment of bacterial infections, including tuberculosis [6,7,8,9,10]. Oximes have also been reported to exhibit a wide range of biological activities, such as anti-inflammatory [11,12,13,14,15] and anti-human immunodeficiency (HIV) agents that can inhibit HIV protease [16,17]. Indeed, the anti-inflammatory activity of some oximes has been reported to be comparable to standard anti-inflammatory drugs, such as indomethacin, diclofenac, and dexamethasone [18,19,20]. On the other hand, the introduction of an oxime group into an appropriate chemical backbone is a reasonable approach for the preparation of cytotoxic agents, and many oxime derivatives have been reported to have therapeutic activity for cancer [2,21,22,23,24,25,26,27] and neurodegenerative disorders [28,29,30].

**Figure 1:**
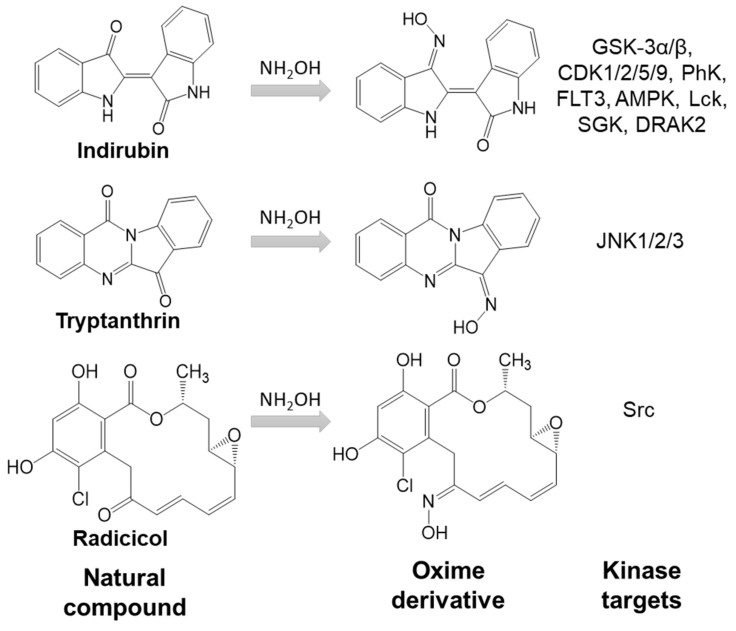
Introduction of oxime groups increases kinase inhibitory activity of natural compounds.

The introduction of oxime groups has been reported to increase the biological activity of several natural compounds (Figure 1). For example, oxime derivatives of gossypol, a natural phenol derived from the cotton plant, exhibit antiviral, insecticidal, and fungicidal activity [31]. Another example is psammaplin A analog, the free oxime group which was responsible for high anticancer activity [32]. Moreover, oxime derivatives of radicicol, a macrocyclic antifungal antibiotic, showed higher inhibitory activity toward Src tyrosine kinase and anticancer activity in comparison with the parent compound [33,34]. Similarly, the oxime modifications made on the biflorin structure led to an increase in antibacterial potential [7]. Acylated oximes derived from triterpenes have shown cytotoxic or antiproliferative activity against many lines of cancer cells [35]. The biological activity of several indirubin oxime derivatives is much higher than that of the plant alkaloid indirubin [36,37]. Finally, we recently reported that the oxime derivative of the natural alkaloid tryptanthrin is a c-Jun N-terminal kinase (JNK) inhibitor [38] (Figure 1).

Oximes have been used in the design of various kinase inhibitors, including phosphatidyl inositol 3-kinase (PI3K) inhibitors [39], phosphorylase kinase (PhK) [40], and JNK [38,41] (see Table 1 and Table 2). For example, indirubin oximes are of interest because of their high affinity binding to the ATP-binding site of protein kinases involved in tumorigenesis, e.g., cyclin-dependent kinases (CDK), glycogen synthase kinase (GSK) 3β, vascular endothelial growth factor receptor 2 (VEGFR-2), c-Src, and casein kinase 2 (CK2) [42,43,44,45,46,47,48]. Many of these kinases are molecular targets for compounds with anticancer activity.

## 2. Chemical Characterization of Oximes

Oxime groups can be easily introduced into organic molecules by reaction of a carbonyl compound (ketone or aldehyde) with hydroxylamine (NH_2_OH) or a hydroxylammonium salt. This chemical modification leads to the appearance of a new pharmacophoric feature, since the oxime moiety contains two H-bond acceptors (nitrogen and oxygen atoms) and one H-bond donor (OH group), instead of the single H-bond acceptor of the C=O group present in the carbonyl precursor. These features, together with the high polarity of oxime groups, can lead to significantly different modes of interaction with receptor binding sites versus the corresponding carbonyl compound, although only small changes occur in the total size and shape of the ligand.

**Figure 2:**
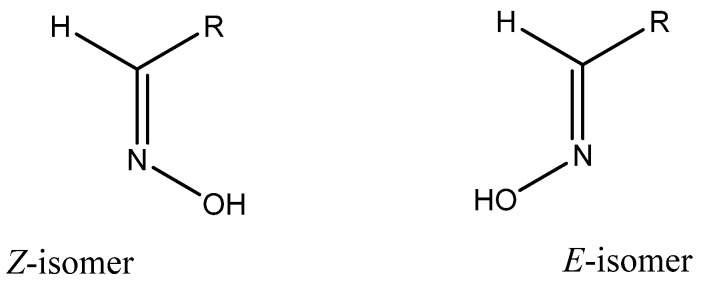
*E*/*Z* isomerism of aldoximes.

Oximes of aldehydes and non-symmetrical ketones can be obtained in two geometrical isomeric forms that are assigned either *E* or *Z* configurations with respect to the C=N bond (Figure 2). For many oximes, the energy barrier for *Z*,*E*-isomerization is high, i.e., the isomers exist as individual compounds at room temperature and under physiological conditions, as demonstrated by NMR spectroscopy [73]. Oxime stereochemistry can be important for pharmacological properties, as demonstrated by the antidepressant fluvoxamine, where only the *E* isomer is active [74]. It should also be noted that nitric oxide (NO) can catalyze *E*/*Z* isomerization of some oximes, most likely by a spin catalytic mechanism [75].

Major plant oximes are amino acid-derived metabolites. It should be noted that the *E* isomers but not the *Z* isomers of plant oximes have high biological activity, including growth regulation, plant defense, pollinator attraction, and plant communication [4].

The hydrogen atom of the oxime OH group can be replaced with alkyl, acyl, or other substituents, and the general synthetic paths for *O*-substituted derivatives (oxime ethers or esters) include direct alkylation or acylation [76]. In addition, oximation of a corresponding aldehyde or ketone precursor by an appropriate *O*-substituted hydroxylamine is widely used (see, e.g., [38,77]). Many biologically active compounds discussed in the present review are actually oxime ethers or esters. For example, compounds **2** and **12** (Table 1) are *O*-acylated oximes, whereas **3**–**5**, **9**, **13**, **16**, **20**–**22**, **24**, and **27** (Table 1 and Table 2) contain an *O*-alkyloxime fragment. The high reactivity of the oxime OH group makes it possible to obtain corresponding salts (oximates). Typical pK_a_ values for aryloximes in aqueous solutions are ~11 [78], but may decrease to 7–8 in some special cases of oximes with stabilized conjugated bases, e.g., bearing pyridinium moieties [79]. Hence, it is expected that oximates are prone to hydrolysis in an organism. Thus, oxime salts can be regarded as pro-drugs with better bioavailability than the parent oximes.

## 3. Anticancer Activity of Oximes

Several kinases shown in Table 1 and Table 2 are potential targets for anticancer therapy, and the anticancer activities of selected oximes in various in vitro and in vivo models are summarized in Table 3 and Table 4.

For example, CDKs are serine/threonine kinases that represent promising therapeutic oxime targets for treating various types of cancer [87]. Likewise, GSK-3 performs critical functions in many cellular processes, such as tumor growth, cell invasion, metastasis, and apoptosis (reviewed in [88,89]). Additional oxime targets include Aurora kinases, which are a group of serine/threonine kinases responsible for the regulation of mitosis. Aurora A and Aurora B are validated anticancer targets, and the development of Aurora kinase inhibitors has progressed from preclinical to clinical studies [90,91]. Oximes can also inhibit FMS-like tyrosine kinase-3 (FLT3), which is recognized as a drug target for the treatment of acute myeloid leukemia (AML), as activating mutations of FLT3 have been found in ~30% of AML patients. Targeted inhibition of FLT3 has shown promising results in the treatment of FLT3 mutation-dependent AML (for review [92]). Janus kinase 1 and 2 (JAK1/2) inhibitors represent an emerging and promising pharmacological class of anticancer drugs used notably for the treatment of some myeloproliferative neoplasms [93]. Finally, dual-specificity tyrosine-phosphorylated and regulated kinase 1A (DYRK1A) is overexpressed in a variety of diseases, including a number of human malignancies such as hematological and brain cancers (for review [94]), and could be an ideal oxime target.

**Figure 3:**
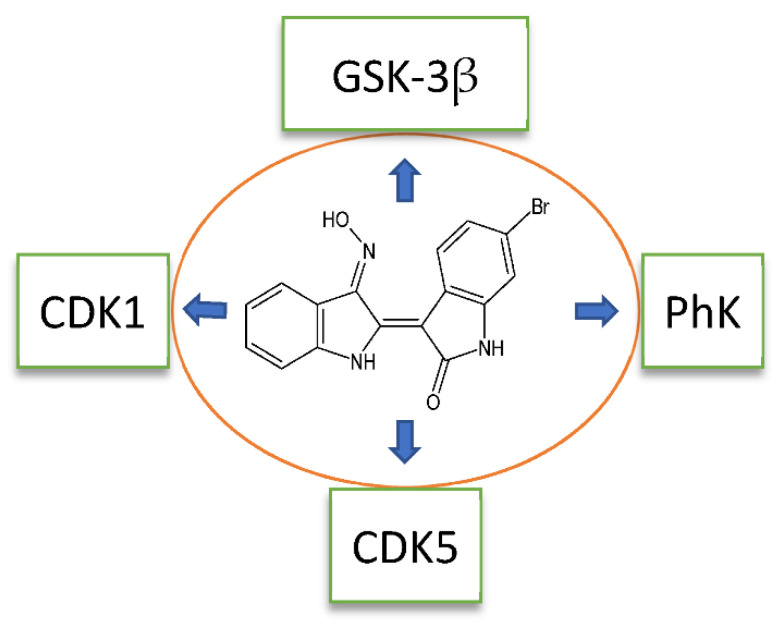
Compound **11** is a multitargeted kinase inhibitor.

Most reported oximes are multitargeted kinase inhibitors (see Table 1 and Table 2 and Figure 3) and can inhibit multiple intracellular signal transduction pathways. Therefore, they have therapeutic advantages over single-targeted inhibitors and have become a focus of antitumor drug research in recent years [95,96]. Recent reviews summarize the mechanisms of action of multitarget therapies and results of the latest clinical trials (e.g., [97,98]). On the other hand, these compounds may exhibit adverse events involving several biological systems due to their mechanism of multitargeted inhibition [99].

## 4. Anti-Inflammatory Activity of Oximes

Several of the kinases targeted by oximes represent potential targets for anti-inflammatory therapy, and the activities of selected oximes in various in vitro and in vivo models of inflammation are summarized in Table 5 and Table 6, respectively. For example, CDKs are potential oxime targets that play regulatory roles in influencing the pro-inflammatory functions of various cytokines during inflammation. CDKs initiate inflammatory responses by triggering the activity of prominent pro-inflammatory transcription factors, such as nuclear factor κB (NF-κB), signal transducer and activator of transcription 3 (STAT3), and activator protein 1 (AP-1) [100]. Likewise, the serine/threonine protein kinase GSK-3β has been implicated as an important regulator of the inflammatory response [101], and contributes to NF-κB activation, as well as to the induction of NF-κB-targeted pro-inflammatory molecules [102]. Indeed, GSK-3β inhibitors have potent anti-inflammatory activity and have been shown to be useful in treating neurodegenerative diseases, including Alzheimer’s disease (reviewed in [103,104]). Finally, recent findings from a Phase 3 clinical trial showed that patients with rheumatoid arthritis who were treated with a JAK1/2 inhibitor had significantly greater improvements in pain reduction [93,105]. Thus, oximes targeting JAK1/2 also have anti-inflammatory potential.

## 5. Indirubin Oxime-Based Kinase Inhibitors

Indirubin, a *bis*-indole alkaloid found in some terrestrial plants and sea shells, is the active ingredient of Danggui Longhui Wan, a traditional Chinese herbal medicine used in the treatment of chronic myelocytic leukemia [25]. Indirubin inhibits various kinases in vitro and is thought to exert its action in vivo by this mechanism [43]. There have been a number of attempts to improve the biological activity and selectivity of indirubin through analog synthesis. Most efforts have focused on modifying this natural product structure by adding substituents around the fused phenyl rings or by converting its 3′-carbonyl group into an oxime [37] (Figure 1). The latter modification was shown to increase the potency of indirubin and its halogenated derivatives toward CDK1, CDK2, CDK5, GSK-3α/β, Aurora kinases A-C, FLT3, and JAK1/2 (reviewed in [36]).

Among the synthetic indirubin analogs, indirubin-3′-monoxime (compound **1**) has been reported to inhibit growth of human MCF-7 breast cancer cells [124,125], osteosarcoma [82], and cholangiocarcinoma cells [81]. Compound **1** also suppressed the production of interleukin (IL)-1β, IL-6, NO, inducible nitric oxide synthase (iNOS), and cyclooxygenase 2 (COX-2) expression via downregulation of NF-κB and JNK signaling pathways in lipopolysaccharide (LPS)-treated murine RAW264.7 macrophage cells [112].

Using a combination of in silico virtual screening of potential anti-diabetic candidates and an in vitro study using an insulin-resistant model of 3T3-L1 adipocytes, Choudhary et al. [106] showed that **1** prevented lipid-induced impairment of the insulin signaling pathway in adipocytes via A2A adenosine receptor activation. While compound **1** reduced lipid-induced adipocyte inflammation by inhibiting NF-κB dependent pro-inflammatory cytokine expression, it also augmented cAMP responsive element binding protein (CREB) activation, favoring an overall anti-inflammatory state [106].

The pathogenesis of atherosclerosis is complex and depends on altered cholesterol metabolism and inflammation [126]. During the onset of atherosclerosis, monocytes adhere to sites of endothelial damage and migrate into the subendothelial layer, where they contribute to early lesion development by accumulating lipids and by secreting cytokines, growth factors, and leukotrienes. Those mediators facilitate further recruitment of immune cells and stimulate migration of vascular smooth muscle cells (VSMCs) from the medial to the intimal layer and, finally, to their proliferation [127,128]. Interestingly, Blazevic et al. [109] found that **1** inhibited leukotriene-mediated VSMC migration. Moreover, **1** inhibited 5-lipoxygenase (5-LO) in cell-based and cell-free assays [109].

Microglia are the resident immune cells of the brain and play a role in the pathogenesis of various central nervous system diseases [129,130,131]. Activated microglia promote neuronal injury through the release of proinflammatory and cytotoxic factors, including tumor necrosis factor (TNF), IL-1β, NO and reactive oxygen species (ROS) [132]. In LPS-induced models of inflammation in cultured rat brain microglia and in organotypic hippocampal slice cultures, compound **1** was shown to inhibit LPS-related hippocampal cell death, decrease the production of TNF, IL-1β, prostaglandin E_2_ (PGE_2_), and ROS, and also reduce LPS-induced NF-κB activation [108]. In addition, compound **1** was also reported to prevent neuronal apoptosis via inhibition of GSK-3β and extracellular signal-regulated kinase (ERK) [111,133]. These results suggest that compound **1** provides neuroprotection by reducing the production of various inflammatory mediators by activated microglia. Likewise, Sathiya Priya et al. [29] reported that compound **1** reduced the levels of NF-κB in nuclear extracts and decreased expression of TNF and IL-6 in a model of neuroinflammation (high fat/high fructose diet in mice). In addition, compound **1** may suppress aberrant NF-κB signaling via inactivation of IκB kinase β (IKK-β), an enzyme that is involved in propagating the cellular response to inflammation. Treatment with **1** significantly decreased the formation of dark neurons, which clearly indicates its recuperative effects towards neuronal apoptosis. Among the anti-apoptotic effects reported for compound **1** are the attenuation of pro-apoptotic Bax and caspase-3 expression, along with an increase in anti-apoptotic Bcl-2 [29].

Compound **1** can inhibit several different kinases, including CDK1, CDK2, CDK5, CDK9, GSK-3α/β, PhK, FLT3, AMP-activated protein kinase (AMPK), Lck, and serum- and glucocorticoid-induced kinase (SGK) [17,37,40,43,44,49,50] (Figure 3). The interaction mode of **1** is similar for both CDK2 and CDK9 [53]. According to reported docking studies, compound **1** forms H-bonds with hinge residue Cys106 via N1′ and O2 atoms. In addition, the oxime moiety is H-bonded via the OH group to the backbone carbonyl group of Ile25 [53]. There is an important link between activation of GSK-3β, amyloid deposition, and neuroinflammation. Indeed, treatment of murine microglial BV-2 cells with compound **1** greatly reduced LPS-stimulated migration, IL-6, and the expression of iNOS and NO production [30]. Likewise, **1** effectively prevented neuronal apoptosis via inhibition of GSK-3β [111,133] and suppression of inflammation, as GSK-3β has been shown to activate NF-κB in LPS-stimulated RAW264.7 macrophages [134] and increase expression of pro-inflammatory genes in LPS-stimulated human monocytic cells and mouse hippocampal slice cultures [30].

RNA-dependent protein kinase R (PKR) plays an important role in inflammation, insulin sensitivity, and glucose homeostasis [135]. For example, treatment of cultured rat cardiomyocytes with high glucose induced a significant increase in PKR, JNK, caspase-3, NF-ĸB, and ROS generation. Notably, all of these inflammatory responses were attenuated by pretreatment with compound **1** [107].

Compounds **1** and **2** (indirubin-3′-acetoxime) have also been shown to be relatively moderate inhibitors of PhK [40,136], which coordinates hormonal and neuronal signals to initiate the breakdown of glycogen. In comparison with indirubin, the parent non-oxime analog, compound **1** forms additional H-bond interactions with Glu110 of the γ catalytic subunit of PhK [136]. Compound **2** has higher PhK inhibitory activity and, in docking experiments, the acetoxime methyl group of **2** partially occupies a space of negative electrostatic potential created by the Glu153 oxygen and the PhK Glu110 and Asp167 side chain carboxylates [40]. These authors performed a thorough quantum mechanics/molecular mechanics (QM/MM) study of PhK-inhibitor interactions and found that the introduction of an oxime or acetoxime moiety in place of the 3′-carbonyl group in the indirubin molecule led to significantly more negative Δ*E*_QM/MM_, indicating more effective binding to PhK due to strong anchoring of the oxime or acetoxime group within a subpocket between Glu110, Glu153, and Asp167 [40].

In the context of the current coronavirus COVID-19 pandemic, the antiviral and anti-inflammatory properties of indirubin oxime derivatives should also be considered. Notably, compound **1** can suppress pro-inflammatory factors associated with viral infection, including chemokine CXCL10 (one of the key factors contributing to lung inflammation during H5N1 influenza virus infection), interferon (IFN)-β, and monocyte chemoattractant protein 1 (MCP-1) [110]. In addition, compound **1** delayed H5N1 virus replication in primary cell culture models [110].

Several different indirubin analogs have been synthesized to improve water solubility and bioavailability. A variety of side chains were introduced at the 3′-position, leading to the synthesis of compounds **3–5** (Figure 1) [46,47,53,124]. Likewise, compound **3**, which contains a dihydroxypropyl 3′-oxime substituent together with an OCH_3_ group, is a potent inhibitor of Src kinase, and it downregulated the constitutively activated signal transducer and activator of transcription 3 (STAT3) or STAT5 in human breast cancer CML cells [46]. Compound **3** also inhibited CDK2, CDK6, CDK16, and GSK-3β [46,52,53]. High-grade gliomas can secrete large amounts of inflammatory cytokines and growth factors that promote autocrine tumor growth. Interestingly, **3** was able to suppress pro-inflammatory genes, including IL-1α, IL-1β, IL-12, prostaglandin endoperoxide synthase 2 (PTGS-2), and Toll-like receptor 4 (TLR4), as well as the secretion of the pro-inflammatory cytokine IL-6 in LN-18 and T98G glioblastoma cells [137]. Similarly, 6-bromoindirubin-3′-glycerol-oxime ether suppressed LPS-induced secretion of IL-1β and PGE_2_ via the inhibition of GSK-3β [138]. Unexpectedly, compound **4** appears to be a strong dual inhibitor of JAK/signal transducer and activator of transcription 3 (STAT3) and Src family of protein tyrosine kinases (SFKs)/STAT3 signaling that is associated with the induction of apoptosis in human pancreatic cancer cells [47,139]. It was also found that **4** is a potent inhibitor of a broad spectrum of serine/threonine and tyrosine kinases, including CDK2, JAK1/2, Tyk2, c-Src, Lyn, Hck, Aurora A, c-Kit, GSK-3β, IGF1R, VEGFR2, and ABL [47,52]. Another important family of synthetic indirubins are the 5-substituted analogs of compound **1**, such as compounds **6**–**8** and **10** (Figure 1). Compound **6** was found to inhibit Aurora kinase A but had no effect on the kinase activities of c-Met, ALK, and JAK2 [55]. Likewise, compound **7** was reported to inhibit CDK2 and induce apoptosis of lung cancer cells [140,141]. The presence of an oxime group was also found to be essential for increasing the inhibitory activity of compound **8** against death-associated protein kinase-related apoptosis-inducing protein kinase (DRAK) 1/2, a serine/threonine kinase belonging to the death-associated protein kinase (DAPK) family [51]. According to docking results, the oxime OH group of compound **8** acts as an H-bond donor with respect to the Glu117 carboxyl oxygen of DRAK. The authors suggested an important role for this interaction in the binding of **8** to DRAK, along with other H-bonds formed by **8** with Glu111 and Ala113 via NH and C=O in the indolin-2-one moiety [51]. Finally, compound **9** and 5-fluoro-indirubin-3′-oxime have been recognized as potent inhibitors of FLT3, which is involved in cancer development, especially leukemia [49,56].

Halogenated indirubins are among the most important subcategories of indirubins, with the main representatives being 6-bromoindirubin and 6-bromoindirubin-3′-oxime (**11**). Notably, the affinity of compound **11** for GSK-3β (IC_50_ = 5 nM) is 100-fold greater than that of 6-bromoindirubin **[44]**. Indeed, the oxime analogs generally exhibit 5–10 times greater inhibitory activity toward GSK-3 β compared to the corresponding non-oxime halogenated indirubin derivatives [142]. Docking of **11** into GSK-3β was reported by Nisha et al. [143], who found that an oxime group forms H-bonds with Val135.

Compound **11** appears to have significant therapeutic potential due to its anti-inflammatory properties. For example, Liu et al. [12] investigated the effects of **11** on inflammatory signaling in mouse mammary epithelial cells (MMECs) and on LPS-induced mastitis in mice [12] and reported that it inhibited the TLR4/NF-κB and TLR4/mitogen-activated protein kinase (MAPK) pathways. This resulted in inhibition of JNK, ERK, and p38 phosphorylation, downregulation of IL-6, IL-1β, TNF, and myeloperoxidase (MPO) expression, and upregulation of IL-10 expression in MMECs. Consequently, compound **11** pretreatment downregulated the expression of the proinflammatory factors IL-1β, IL-6, TNF, and MPO in mammary glands and reduced inflammatory lesions in breast tissue of LPS-injected mice [12]. Similarly, Park et al. [113] showed that the inhibition of GSK-3β activity by **11** delayed the inhibitor of nuclear factor κB (IκBα) degradation and diminished expression of TNF in LPS-stimulated neutrophils and macrophages. In addition, compound **11** blocked GSK-3β phosphorylation/activation, decreased the levels of the proinflammatory cytokines TNF, IL-1β, and IL-6, elevated the level of anti-inflammatory cytokine IL-10, inhibited microglia activation and cell apoptosis, and improved the sensorimotor deficits of rats after intracerebral hemorrhage [118].

Kwon et al. [13] showed that compound **11** inhibited the NF-κB, JNK, c-Jun, activating transcription factor (ATF)-2 and p38 pathways in fibroblast-like synoviocytes (FLS). Consequently, **11** treatment also diminished the production of proinflammatory mediators IL-1, IL-6, MCP-1, MCP-3, COX-2, and matrix metalloproteinase (MMP)-9 by these FLS. The anti-inflammatory effects of compound **11** were also evaluated in vivo in a mouse model of collagen-induced arthritis (CIA). Treatment of CIA mice with **11** attenuated clinical and histological signs of arthritis. For example, infiltration of T cells, macrophages, and tartrate-resistant acid phosphatase positive cells was decreased in joint sections of mice with arthritis. Likewise, serum levels of IL-1β, IL-6, TNF, and IFN-γ were inhibited by compound **11** treatment [13]. Similarly, **11** inhibited production of IFN-γ and nuclear translocation of T-box (Tbx21), a transcription factor of IFN-γ, in CD3^+^ T cells in mouse model of skin inflammation [117]. In addition, this treatment attenuated epidermal hyperproliferation and dermal angiogenesis [117]. Compound **11** has also been shown to inhibit periodontal inflammation, promote bone regeneration, and induce the expression of bone-forming markers in a mouse periodontitis model [120].

Ischemic stroke triggers blood–brain barrier (BBB) breakdown via destabilization of the tight junctions and deregulation of the transport mechanisms [144]. Subsequently, BBB breakdown can contribute to the progression of secondary brain injury by causing edema formation, increasing the accumulation of toxic metabolites, and exacerbating the inflammatory response [145]. Another consequence of BBB disruption can be hemorrhagic transformation, which is a major complication of ischemic stroke, causing significant morbidity and mortality in patients [146]. The formation and maintenance of the BBB is ensured by correct functioning of the Wnt/β-catenin pathway [147]. Interestingly, compound **11** can induce Wnt/β-catenin pathway activation and reduce the incidence of hemorrhagic transformation associated with delayed recombinant tissue plasminogen activator (rtPA) administration [119]. Specifically, compound **11** treatment was shown to limit BBB breakdown via the promotion of tight junction formation and repression of endothelial basal permeability, independently of rtPA proteolytic activity. The effects of **11** on tight junctions was apparently due to is ability to stabilize β-catenin in the cytosol and stimulate its subsequent translocation to the nucleus. As a consequence, compound **11** treatment decreased brain edema, reduced IgG extravasation, and diminished the incidence of perivascular petechial bleeding 24 h after middle cerebral artery occlusion [119].

A newer area of compound **11** investigation is focused on aging. Liver aging is associated with age-related histopathological and functional changes that significantly enhance the risk of numerous diseases or disorders developing in elderly populations. Studies have demonstrated that **11** can reduce oxidative stress, improve lipid metabolism, enhance autophagy, and significantly reduce liver aging via modulation of the GSK-3β and mTOR pathways [121].

Compounds **12**–**15** are brominated indirubin derivatives, and **12** has been reported to be a potent inhibitor of GSK-3α/β and PhK [40,44]. Likewise, compound **13** exhibited inhibitory activity toward c-Src, JAK1, JAK2, and TYK2 [60]. In contrast, the 7-bromoindirubin-3′-oxime (**14**) was found to be a selective inhibitor of Aurora C [57]. 6-Bromoindirubin-3′-[*O*-(2-piperazine-1-ylethyl)] oxime has also been reported to inhibit proinflammatory pathways, including GSK-3α/β [59]. Finally, 5′-carboxylate derivative **15** was reported to inhibit DYRK1a and DYRK2 with enhanced selectivity [58].

## 6. Miscellaneous Oxime Group-Containing Kinase Inhibitors

The structures of oxime kinase inhibitors with non-indirubin scaffolds are shown in Table 2. These inhibitors were designed to inhibit various kinases, including vascular endothelial growth factor receptor 2 (VEGFR-1/2/3), B-Raf, ErbB1/2/3, PI3K isoforms α, β, γ, σ, and γ. Radicicol is a naturally occurring macrocyclic antifungal agent. Interestingly, oximation of radicicol increases its inhibitory activity towards Src [34], a tyrosine kinase that can regulate a number of signaling pathways impacting tumor cell behavior, including proliferation, survival, migration, invasion, and angiogenesis [148]. Similarly, the radicicol oxime derivative **17** had even higher anticancer activity than radicicol [33]. Raf isoforms are activated by phosphorylation via downstream regulation from the MAPK pathway. For example, B-Raf kinase plays a significant role in healthy cell growth by regulating B-Raf activity, and B-Raf mutations can lead to the development of cancer and other diseases [149]. Indeed, the oxime **18** is a highly selective, potent, and orally bioavailable B-Raf inhibitor with anticancer activity [62,150]. The major ketone metabolite of compound **18** is inactive [151], strongly suggesting that that the oxime group is responsible for kinase inhibitory activity. Likewise, oxime **19** was identified by Takle et al. [63] as another potent inhibitor of B-Raf.

**Figure 4:**
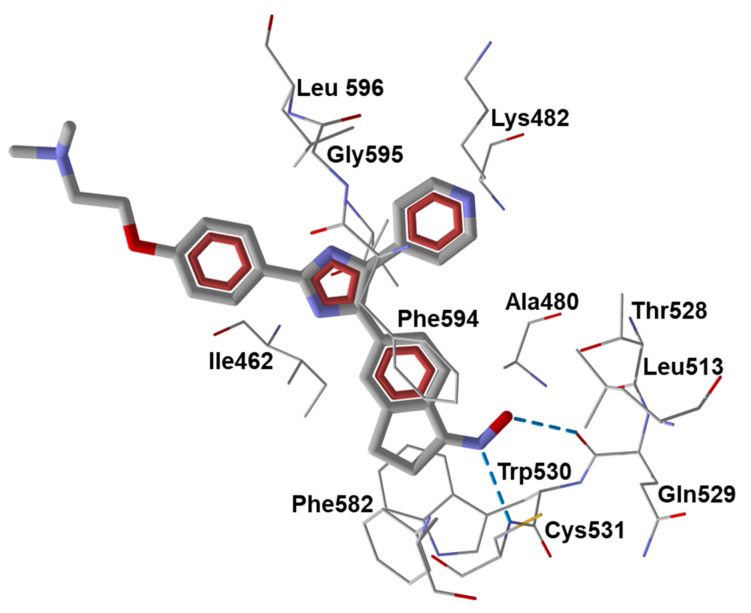
Docking pose of compound **19** (*Z*-isomer) in B-Raf (PDB: 1UWH). Amino acid residues within 3 Å from the pose are visible. H-bonds are shown in dashed blue lines. H-bond lengths with Gln529 and Cys531 are equal to 1.73 and 1.86 Å. They are formed with participation of oxime OH group and nitrogen atom, respectively.

To evaluate the role of the oxime group in binding, we conducted additional molecular docking of compounds **18** and **19** towards B-Raf (PDB: 1UWH). Our modeling experiments showed that the best docking pose of **18** with B-Raf structure forms a strong H-bond to Cys531 with participation of the pyridine nitrogen atom (Figure 4). In addition, a weaker H-bond is formed between the OH group of the 2-hydroxyethyl moiety and Phe594, while the oxime group has non-valent attractive interactions with Leu504, Ile526, and Thr528. The partial docking score for the =N-OH moiety of compound **18** is −8.04 kcal/mol. We found that the inactive ketone metabolite of **18** has a similar docking pose, with the pyridine nitrogen atom H-bonded to Cys531, while the dihydroindene moiety is rotated about the exocyclic C-C bond. In this conformation, the ketone oxygen atom has much weaker Van der Waals interactions with Lys482, Ile526, and Thr528 (partial docking score is −2.52 kcal/mol). Compound **19** has a bulky imidazole ring, and is bound to B-Raf, with the dimethylamino tail directed outwards from the kinase cavity (Figure 4). However, strong H-bonding is present between the oxime nitrogen atom and Cys531, and the oxime OH group forms an H-bond with Gln529.

Vasculature development is believed to be dependent on VEGF and its receptor tyrosine kinases, mainly VEGFR-2 and the angiopoietins (Ang-1 and Ang-2) and their receptor tyrosine kinase (primarily TIE-2). Thus, optimal antiangiogenic kinase therapy may require concurrently blocking both TIE-2 and VEGFR-2 signaling to inhibit tumor growth and metastasis. Compound **20** was reported to be a potent VEGFR-2 tyrosine kinase inhibitor [64] and, according to our results of docking into VEGFR-2 (PDB: 1YWN), is H-bonded to the NH group of Cys917 via its carbonyl moiety. The substituted oxime group =N-O- does not form H-bonds with the enzyme, although it has attractive Van der Waals interactions, mainly with Cys1043 and Asp1044. The partial docking score for the oxygen and nitrogen atoms of the oxime group is −9.11 kcal/mol. Compound **20** inhibited VEGF-dependent proliferation of human vascular endothelial cells and markedly regressed tumors in an A549 lung cancer xenograft model [64]. Compound **22** was also identified as a potent and selective inhibitor of VEGFR-2. This oxime also inhabited the closely related tyrosine kinases, Ret and Kit, but had no significant activity against VEGFR-1 or VEGFR-3 [66]. Notably, treatment of nude mice bearing human A431, HCT116, and A375 tumors with compound **22** resulted in up to 90% tumor growth inhibition [66].

Fused dihydroindazolopyrrolocarbazole oximes have been identified as low nanomolar dual TIE-2 and VEGFR-2 receptor tyrosine kinase inhibitors, with the most potent being compound **27**. This compound inhibited VEGF-induced human umbilical vein endothelial cell (HUVEC) capillary-tube formation and was orally active in an A375 human tumor xenograft melanoma model with no observed toxicity [70].

Checkpoint kinase 1 (Chk1) and epidermal growth factor receptor (EGFR) are therapeutic targets for treatment of acute and chronic leukemias [152] and high-grade serous ovarian cancer [153,154]. Thus, it is significant that compounds **23**, **25**, and **27** have been reported as Chk1 and EGFR tyrosine kinase inhibitors [67,69].

Signaling pathways regulated by PI3Ks have been shown to play a role in cancer development and progression. Thus, therapeutic targeting of PI3K has been considered as a possible strategy for treating several types of cancer, including gastrointestinal cancer [155]. For example, compound **24** inhibited PI3Kγ and IL-6 release by concanavalin A-simulated mouse lymph node cells [68]. Similarly, several chromeno [4,3-*c*]pyrazol-4(2*H*)-one oxime derivatives have been shown to target PI3Ks, including PI3Kα, which is inhibited by compound **26**. This compound also exhibited the most potent antiproliferative activity against human colorectal carcinoma HCT-116 cells [39].

CK2 is a ubiquitously expressed and highly conserved serine/threonine or tyrosine kinase that regulates diverse signaling pathways responsible for cell proliferation and apoptosis via interactions with over 500 known substrates. CK2 also plays an extrinsic role in cancer stroma or in the tumor microenvironment [156]. Thus, it is significant that compound **29** can inhibit CK2 kinase with moderate potency [71].

JNKs play important roles in many pathological processes, including autoimmune inflammatory disorders such as rheumatoid arthritis [157]. A number of JNK inhibitors with anti-inflammatory properties have been developed [158], yet few have been developed for the treatment of rheumatoid arthritis. Recently, we reported that 11*H*-indeno[1,2-*b*]quinoxalin-11-one oxime (compound **30**), its sodium salt **IQ-1S**, and tryptanthrin-6-oxime (compound **31**) were JNK inhibitors [41,116]. We found that the side chain oxime substituent was critical for JNK binding and biological activity of these compounds [38,41].

**Figure 5:**
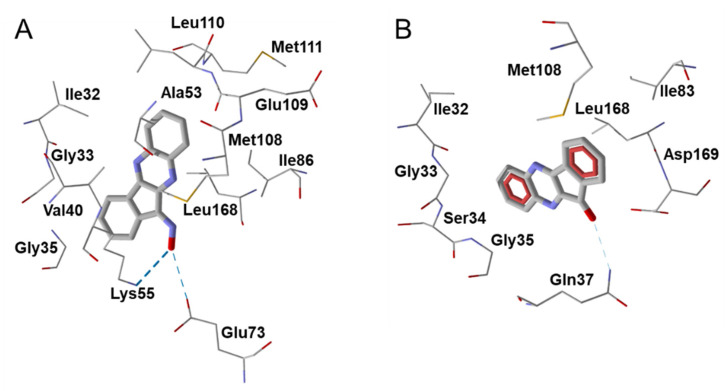
Modeling H-bond interactions of oxime **30** (**A**) and its inactive ketone derivative **IQ-18** (**B**) in the JNK1 binding site (PDB code 1UKI). Residues within 3 Å from the pose are visible. H-bonds are shown as dashed blue lines. Compound **30** forms H-bonds with Lys55 and Glu73. These H-bonds have lengths of 2.08 and 2.58 Å and are formed with the participation of oxime oxygen and hydrogen atoms, respectively. In contrast, **IQ-18** forms one very weak H-bond with Gln37 (calculated length of 2.76 Å).

Molecular modeling studies suggested that H-bonding interactions with participation of the oxime group play an important role in the JNK inhibitory activity of compounds **30** and **31**. In support of this conclusion, the inactive ketone of **30** (**IQ-18**) formed one weak H-bond with Gln37 of JNK1, whereas the oxime group of **30** formed two stronger H-bonds with Lys55 and Glu73 (Figure 5). Similarly, the high JNK inhibitory activity of compound **30** could be modulated by H-bonding interactions with Asn152, Gln155, or Met149 in the JNK3 binding site [41]. Compound **30** inhibited matrix metalloproteinase 1 and 3 (MMP1/3) gene expression induced by IL-1β in human FLS, and significantly attenuated development of CIA [122]. Treatment with **30** either before or after induction of CIA resulted in decreased clinical scores, and joint sections from compound **30**-treated CIA mice exhibited only mild signs of inflammation and minimal cartilage loss compared with those from control mice. Collagen II-specific antibody responses were also reduced. Compound **30** treatment also suppressed proinflammatory cytokine and chemokine levels in joints and lymph node cells [122].

The docking pose of compound **31** was also characterized by strong H-bonding between the oxygen atom of the amide group and the Met111 of JNK1. This compound was H-bonded with JNK2 through its oxime group with Gly171. Finally, **31** was anchored in the JNK3 cavity via H-bonding of the oxime group with Asp207 [38]. Compound **31** demonstrated high binding activity toward all three JNK isoforms (JNK 1-3) [38], inhibited MMP-3 gene expression in IL-1β-stimulated human FLS, and inhibited IL-1β-induced secretion of MMP-1/3 by FLS and synovial SW982 cells and IL-6 by FLS, SW982 cells, HUVECs, and monocytic THP-1 cells [116]. Evaluation of the therapeutic potential of compound **31** in vivo in murine arthritis models showed that it attenuated the development of CIA and collagen-antibody-induced arthritis (CAIA). Collagen II-specific antibody levels were reduced in compound **31**-treated CIA mice. This compound also suppressed the production of proinflammatory cytokines IL-17A, granulocyte-macrophage colony-stimulating factor (GM-CSF), and receptor activator of nuclear factor-κB ligand (RANKL) by lymph node cells from CIA mice [116].

JNK-mediated signaling pathways also play an essential role in cerebral and myocardial ischemia/reperfusion injury [159], and the neuroprotective activity of oxime **30** has been demonstrated in models of focal cerebral ischemia in mice [160] and rats [123], as well as in a model of total cerebral ischemia in rats [161]. Compound **30** inhibited JNK activity in the hippocampus and protected against stroke injury, reduced the infarct size, and limited the neurological deficit of rats after focal ischemia/reperfusion. After global ischemia/reperfusion, **30** decreased the number of animals with severe neurological deficit, increased density of the pyramidal neurons in the hippocampal CA1 area, improved the cerebral microcirculation, and attenuated the endothelial dysfunction. In addition, compound **30** treatment resulted in decreased systolic blood pressure, mean arterial blood pressure, and total peripheral resistance in spontaneously hypertensive rats [114]. Overall, the antihypertensive effects of compound **30** may be due to a combination of the inhibition of myocardial and aorta remodeling, attenuation of blood viscosity due to hematocrit decrease, vasodilatory effects, and decreased endothelin-1 production by the endothelial cells.

## 7. Oximes with Non-kinase Targets

While most of the identified oxime targets have been various kinases, there are some oximes that also have non-kinase targets of action. These targets include 5-lipoxygenase (5-LO), proteases, phosphodiesterase, chemokine receptors, growth factor receptors, and various channels (Table 7). For example, several indirubin oximes, such as compounds **1** and **11**, have been reported to inhibit 5-LO [162], which is required for leukotriene synthesis. Replacement of the 3′-oxime in **1** by a keto group, 3′-methoxime or acetoxime resulted in loss of 5-LO inhibitory activity, indicating that a free oxime moiety in the 3′-position and a hydrogen in position N1 are required for effective inhibitory activity [162]. Additionally, newer derivatives of oleanolic acid oxime, and particularly their conjugates with acetylsalicylic acid, have been shown to downregulate the expression of cyclooxygenase 2 (COX-2) in human hepatoma HepG2 cells by modulating NF-κB signaling [163]. A reduction in COX-2 leads to reduced prostaglandin synthesis, which also inhibits inflammation in a similar fashion to other nonsteroidal anti-inflammatory drugs (NSAIDs).

16α,17β-Epoxypregnenolone-20-oxime was reported to inhibit LPS-induced JNK phosphorylation, iNOS expression, and NO production in BV-2 microglial cells and RAW264.7 macrophages [164,165]. Likewise, the introduction of an oxime at position *12* of dehydroabietic acid, an aromatic abietane-type diterpenoid, increased its anti-proliferative and anti-inflammatory activities in pancreatic cancer Aspc-1 cells [166]. Moreover, a kinase profiling study showed that dehydroabietic oxime had modest inhibitory activity for p90 ribosomal S6 kinase 2 (RSK2) [166], a kinase that has been implicated in cellular invasion and metastasis [166,167,168]. In addition, Chen et al. [167,168] found that oxime derivatives of furo[2,3-*b*]quinolines were more potent than their respective ketone precursors for their ability to inhibit mast cell and neutrophil degranulation, as well as neutrophil ROS production. The precise targets of these oximes have not been identified.

Pillai et al. [178] synthesized a series of tetra-substituted thiophenes and reported that they had anti-inflammatory activity in a carrageenin-induced rat paw edema model [178]. They also found that compounds with aliphatic oxime esters attached with a ketone bridge to the thiophene had higher anti-inflammatory activity than the aromatic oximes. These oxime analogs were also weak to moderate free radical scavengers; however, a direct correlation between anti-inflammatory activity and free radical scavenging activity was not seen [178]. Nevertheless, the authors suggested that these oximes could have potential as anti-inflammatory agents. Likewise, 2-phenylindole-3-carboxaldehyde oxime was reported to inhibit NO production in RAW 264.7 macrophage cells, as well as NF-κB inhibition in human embryonic kidney cells 293 [179]. In addition, oxime derivatives of β-acetoxy-17β-hydroxy-androst-5-ene, such as 3β-acetoxy-androst-5-ene-17 oxime, were shown to have anti-inflammatory activity in a mouse model of ear inflammation [18]. Other steroidal oximes, such as 22-oxocholestane oximes, that were also evaluated as anti-inflammatory agents in the acute ear inflammation model exhibited anti-inflammatory activity [20]. The most active oximes downregulated NF-κB and inhibited expression of pro-inflammatory genes TNF, COX-2, and IL-6, and reduced ear-induced inflammation and edema. Notably, the activity of these oximes was comparable to the potent anti-inflammatory agent dexamethasone [20]. Similarly, (*Z*)-(2-carbethoxyamino-4-methyl-1,3-thiazol-5-yl)-(4-methylphenyl)methanone oxime exhibited anti-inflammatory activity in acute and chronic inflammatory models of rat paw edema [180]. Likewise, the adamantane-containing molecules *O*-(α-acetoxy-benzeneacetyl)-2-tricyclo[3.3.1.13,7]decan-2-one oxime and *O*-(α-propoxy-benzeneacetyl)-2-tricyclo[3.3.1.13,7]decan-2-one oxime) had anti-inflammatory activity comparable to that of diclofenac in a mouse paw edema model [19]. Finally, oral dosing with (*E*)-1-(4-((1*R*,2*S*,3*R*)-1,2,3,4-tetrahydroxybutyl)-1*H*-imidazol-2-yl)ethanone oxime resulted in a decrease in circulating lymphocytes, decreased hind limb swelling, and reduced circulating anti-type II collagen antibodies in a CIA mouse model of rheumatoid arthritis [181].

Human neutrophil elastase (HNE) and proteinase 3 (Pr3) also represent potential oxime targets for the development of anti-inflammatory therapeutics to treat adult respiratory distress syndrome, autoimmune disorders, and hypersensitivity reactions [182,183]. For example, 2-aminobenzaldehyde oxime analogs such as compound **32** were found to have dual inhibitory effects on HNE and Pr3 [15]. This compound was slightly more potent than the commercial HNE inhibitor Sivelestat, which is used in Japan and Korea for the treatment of acute lung injury associated with systemic inflammation [184]. In mouse models of inflammation, treatment with **32** reduced paw edema and acute lung injury [15].

Oxime-based phosphodiesterase (PDE) 4 inhibitors are also being evaluated as potential anti-inflammatory agents, as they have the ability to inhibit the production of inflammatory mediators and cytokines [185]. Several oxime derivatives of rolipram, an inhibitor of PDE4, have been reported to inhibit TNF production in LPS-stimulated RAW264.7 macrophages with higher potency than rolipram [186]. Interestingly, the *E*/*Z*-geometry of oxime was important for activity of these compounds, with *cis*-isomers being more active than the corresponding *trans*-isomers [186].

Several oximes target receptors or ion channels. Among the oxime receptor targets are chemokine receptors, kainate receptors, and growth factor receptors. For example, compound **33** has been reported to be an orally bioavailable, small molecule antagonist of CCR5. Indeed, this compound exhibited potent antiviral activity against HIV-1 infection in vitro and in vivo [169,170]. Another oxime derivative, compound **34** was reported to be a low-affinity inhibitor of the ionotropic kainite receptor GluR_6_, and treatment with **34** was reported to attenuate inflammation-induced thermal hyperalgesia [171,172]. Compound **34** has also been proposed to inhibit neurotoxic effects of kainate receptor agonists in murine cultured cortical neurons [187]. Finally, El-Sherief et al. [188] synthesized a series of oximes with a 1,2,4-triazole scaffold. Some of these oxime hybrids had higher anti-proliferative activity than their corresponding ketones [188], and were determined to be epidermal growth factor receptor (EGFR) inhibitors, as well as moderate inhibitors of B-Raf and tubulin.

Among the oxime channel or transporter targets are transient receptor potential (TRP) channels, acid-sensing channels, and mitochondrial transition pores. For example, compound **35** has been reported to be a potent transient receptor potential ankyrin 1 and vanilloid 1 (TRPA1 and V1) channel antagonist [14]. Similarly, compounds **36** and **37** were found to be selective TRPA1 channel blockers [173,174]. These compounds represent promising new candidates for drug development focusing on neuropathic pain, migraine, and arthritis. Compound **38** is an acid-sensing ion channel (ASIC) blocker with specificity for ASIC1a and ASIC3. This oxime compound reduced pathophysiological nociceptive behaviors in complete Freund’s adjuvant-inflamed and reversed mechanical hypersensitivity in a rat chronic constriction injury model [175]. Interestingly, **38** had no adverse effects on motor function, which are major problems with morphine-based analgesics.

Compound **39** (cholest-4-en-3-one, oxime) is a neuroprotective and neuroregenerative compound that has been reported to rescue motor neurons from axotomy-induced cell death and promote nerve regeneration following sciatic nerve crush in vivo [176]. This compound is thought to bind to two components of the mitochondrial permeability transition pore, the voltage-dependent anion channel (VDAC) and translocator protein, and inhibit pore opening and reduce neuronal apoptosis [176]. The authors suggested that **39** may have therapeutic potential for amyotrophic lateral sclerosis (ALS).

Although most of the known oximes exhibit anti-inflammatory activity, oxime **IMR-23** has been reported to exhibit pro-inflammatory activity in J774A.1 cells and in a mouse model [189], and has been suggested to have potential in the development of adjuvants. Specifically, treatment with **IMR-23** induced the release of pro-inflammatory cytokines IL-1β, IL-6, and TNF, induced the production of antibodies, and led to the generation of antigen-specific T cells [189].

## 8. Metabolism of Oximes and NO Production

Metabolism of oximes catalyzed by cytochrome P450 can lead to release of NO [190,191,192,193]. For example, oxidative breaks of the oxime C=N bond and the formation of a C=O bond lead to the transfer of one oxygen atom from O_2_ to the compound and simultaneous release of NO [194]. For various oximes, it has been reported that this reaction proceeds in liver microsomes with the participation of cytochromes P450, NADPH, and O_2_ [190,191,195,196]. The participation of cytochromes P450 is confirmed by the fact that inducers and inhibitors of microsomal oxidation can activate or inhibit oxidative metabolism of oximes, respectively [190,195]. For acetoxime, it was shown that ROS play a key role in oxidation of the compound to NO by liver microsomes [191]. Jousserandot et al. [196] described a mechanism for such oxidative cleavages of oximes with formation of nitrogen oxides by cytochrome P450, with the involvement of O_2_^•−^ and its Fe-complexes [(FeIII-O_2_^−^), or (FeII-O_2_)] as the main reactive species. Amidoximes oxidized together with NO also release NO-related products, such as NO_2_^−^ and NO_3_^−^ [190,191]. For example, the rate of arylamidoxime microsomal oxidation of para-hexyloxy-benzamidoxime rapidly decreases with time, which is related to the inactivation of cytochromes by the formation of P450-Fe(II)-NO and P420-Fe(II)-NO complexes [190].

**Figure 6:**
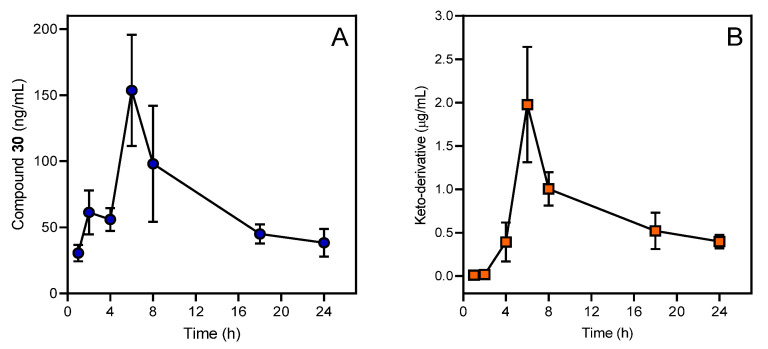
Pharmacokinetic profile of compound **30** (**A**) and its keto-derivative **IQ-18** (**B**) in blood plasma of SD rats after a single intragastric administration of compound **30** at a dose of 50 mg/kg. Mass spectrometric analyses were performed using a Shimadzu LC-20 (Kyoto, Japan), coupled with an ABSCIEX API 3200 triple quadrupole mass spectrometer (USA).

Microsomal oxidation of amidoximes to the corresponding nitriles, and of ketoximes to the corresponding nitroalkanes, are not inhibited by superoxide dismutase (SOD), and are performed by a cytochrome P450 active species, presumably the high-valent P450-Fe-oxo complex. In contrast, microsomal oxidation of amidoximes to the corresponding ureas and amides was also found to be mainly performed by O_2_^•−^, as shown by the inhibitory effect of SOD and the ability of the xanthine-xanthine oxidase system to give similar oxidation products [196]. Further steps in the metabolism of keto-derivatives and their excretion from the organism will depend on the specific structure of the aryl ring. For example, Figure 6 shows the pharmacokinetic curves of compound **30** and its keto-derivative.

The vasodilator effects of oximes on isolated vessels with denuded endothelium and endothelium [161,190,197,198] substantiated the existence of other (non-microsomal) pathways of oxime biotransformation and the production of NO. Treatment with formamidoxime, acetaldoxime, acetone oxime, acetohydroxamic acid, or formaldoxime resulted in a relaxation of rat endothelium-denuded rings [198]. Neither inhibitors of NO synthases nor inhibitors of cytochrome P450 reduced the vasodilator effect of oxime derivatives. Furthermore, inhibition of the vasodilatory effects of these oximes under the influence of 7-ethoxyresorufin suggests the possibility of the participation of NAD(P)H-dependent reductases in the NO-donating properties of oximes [190,197,198]. For a broader review on the biological pathways of amidoximes, see [193]. NO is involved in many physiological processes, such as neurotransmission, blood pressure regulation, and immune modulation. However, in some diseases, such as hypertension and diabetes, the ability of endothelial NO synthase (eNOS) to generate NO is impaired [6,199]. For this reason, compounds capable of being oxidized to release NO in pathways other than NOS are of high interest. Indeed, NO donors have been reported to exhibit anti-inflammatory and anticancer activities [200,201]. For example, NO-donating NSAIDs, which are safer than their NSAID counterparts, inhibit the growth of colon cancer cells with greater potency than traditional NSAIDs [202]. Due to their NO-donating capacities, some oxime derivatives have also been shown to offer therapeutic potential for the treatment of erectile dysfunction, as well as cardiovascular diseases [203,204]. Likewise, a number of oxime derivatives have been shown to exhibit antithrombogenic, hypotensive, and cardiotonic activity [198,205,206]. For example, amidoximes and oximes have been shown to inhibit platelet aggregation, decrease thrombus formation, induce vasodilation, and lower intraocular pressure [192,197,199,207,208,209]. NO-donating oxime hybrids also have been reported to have gastroprotective activity versus their corresponding ketone precursors, which also may be attributed to the release of NO [210].

## 9. Conclusions and Perspectives

Oxime groups have been successfully introduced into a large number of therapeutic leads for the development of kinase inhibitors with anticancer and anti-inflammatory activities. The kinase selectivity of oximes does not appear to be due to the oxime group. Rather, selectivity seems to be due to the scaffold of the molecule, since some oximes are highly selective (e.g., JNK inhibitors **30** and **31** [38,41]), while others, such as indirubin, have a wide spectrum of kinase targets. In this regard, compounds **30** and **31** are of particular interest as candidates for the development of new anti-inflammatory drugs, since they are highly selective for JNKs.

While the presence of a terminal oxime group is necessary for the activity of these compounds, the oxime group also offers a significant advantage in drug design versus carbonyl groups because of the presence of two H-bond acceptors (N and O atoms) and one donor (OH group). Additionally, the metabolism of oximes can lead to the release of NO, which may also be therapeutically beneficial [56]. The important role of the oxime group is supported by docking results revealing direct participation of oxime moiety in interactions with kinase binding sites. On the other hand, there has been some concern regarding the development of new drugs based on oxime derivatives. For example, a disadvantage of compound **11** and other indirubin derivatives is the high affinity of indirubin for ATP-binding pockets and the high degree of similarity between ATP cavities within the serine/threonine and tyrosine kinases, leading to multi-targeting. However, single molecules targeting two (or three) kinases is considered less problematic for current pharmaceutical development, and **11** is considered to have significant potential as a therapeutic for treatment of inflammatory and degenerative diseases. One major unsolved issue related to oxime derivatives is their unfavorable physicochemical properties, including poor solubility and membrane permeability, which results in low plasma bioavailability and a short half-life that limits their suitability as drugs [211,212]. However, compounds **1** and **30** can apparently cross the BBB easily, suggesting that these oximes might be useful for treating brain disorders. New approaches are being developed to improve oxime PK/PD parameters [213,214,215]. For example, complexing oxime molecules into a dendrimer carrier has been proposed as a strategy to extend their plasma duration through a mechanism of release kinetics, so that loaded drug molecules are released over a longer half-life. Choi et al. [215] demonstrated that drug-dendrimer complexes form in a specific manner, wherein each oxime molecule interacts through electrostatic attraction with the primary amine terminated at the peripheral branch of the dendrimer [215]. The importance of the oxime group in kinase binding suggests that additional introduction of this group in the structures of known kinase inhibitors could improve their potency. In addition, oximes with non-kinase targets could be screening toward a broad kinase panel for identification of novel kinase inhibitors.

It is important to note that most of the oximes reviewed here were discovered during compound optimization and not high-throughput screening (HTS). In addition, most of these compounds were characterized in cell-free enzymatic systems and supported in independent test systems. Although compound **30** was originally discovered using HTS in a cell-based assay, the target of this compound was verified using multiple enzymatic assays, cell-based assays, structure–activity relationship (SAR) analysis, and animal experiments. Based on this compound and the absolute requirement for the oxime group in JNK inhibitory activity, we also developed compound **31**, which was also validated in cell- and enzyme-based assays and in animal experiments. Thus, it is unlikely that these compounds or the oximes reviewed here are pan assay interference compounds (PAINS) [216,217]. Nevertheless, this is an important consideration in small molecule screening and will need to be addressed as oximes are developed for new therapeutics.

## Figures and Tables

**Table 1:**
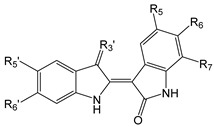
Indirubin oxime-based kinase inhibitors and their kinase targets.

Compound	R_5_	R_6_	R_7_	R_3′_	R_5′_	R_6′_
**1**	H	H	H	=N-OH	H	H
**2**	H	H	H	=N-OAc	H	H
**3**	H	H	H	=N-OCH_2_CHOHCH_2_OH	H	H
**4**	OCH_3_	H	H	=N-O-CHOH-CH_2_OH	H	H
**5**	OCH_3_	H	H	=N-O-(CH_2_)_2_OH	F	F
**6**	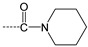	H	H	=N-OH	H	H
**7**	NO_2_	H	H	=N-OH	OH	H
**8**	NHC(O)Bu	H	H	=N-OH	H	H
**9**	C(O)OCH_3_	H	H	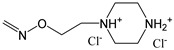	H	H
**10**	I	H	H	=N-OH	H	H
**11**	H	Br	H	=N-OH	H	H
**12**	H	Br	H	=N-OAc	H	H
**13**	H	Br	H	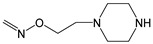	H	H
**14**	H	H	Br	=N-OH	H	H
**15**	H	H	Br	=N-OH	COOH	H
**16**	F	H	H	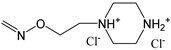	H	H
**Compound**	**Kinase target (IC_50_, μM)**				**Ref.**	
**1**	GSK-3α/β (0.022), CDK1 (0.18), CDK2 (0.7), CDK5 (0.1), CDK9 (2.4), PhK (0.21), FLT3 (0.033), AMPK (0.22), Lck (0.3), SGK (0.38), DRAK2 (0.71)				[37,40,43,44,49,50,51]	
**2**	CDK1 (1.2), CD5 (0.7), PhK (0.17), GSK-3α/β (0.2)				[40]	
**3**	CDK2 (0.23), Src (0.43), CDK6, CDK16, GSK-3β				[47,52]	
**4**	CDK2 (0.043), JAK1 (0.01), JAK2 (0.074), Tyk2 (0.001), c-Src (0.011), Lyn (0.03), Hck (0.264), Aurora A, c-Kit, GSK-3β, IGF1R, VEGFR2, ABL				[47,52]	
**5**	CDK2 (0.4), CDK9 (0.3)				[53]	
**6**	Aurora A (0.37)				[54]	
**7**	CDK2 (0.002)				[55]	
**8**	DRAK2				[51]	
**9**	FLT3 (0.003), JAK2 (0.52), JAK3 (0.69), cMET (0.24), IRAK4 (0.3)				[56]	
**10**	GSK-3α/β, CDK1, CDK5				[37]	
**11**	GSK-3β (0.005), CDK1 (0.32), CDK5 (0.083), PhK, Aurora A (0.6), Aurora B (0.9), Aurora C (0.2), DYRK1a (1.7), DYRK2 (2.1)				[40,44,57,58]	
**12**	CDK5 (2.4), GSK-3α/β (0.01), PhK (0.33)				[40,44]	
**13**	c-Src (0.0002), JAK1 (0.6), JAK2 (0.03), TYK2 (0.05), GSK-3β (0.003)				[59,60]	
**14**	Aurora B (4.6), Aurora C (0.7), DYRK1a (1.9), DYRK2 (1.3)				[57,58]	
**15**	DYRK1a (0.21), DYRK2 (0.13)				[58]	
**16**	FLT3 (0.001)				[61]	

**Table 2:**
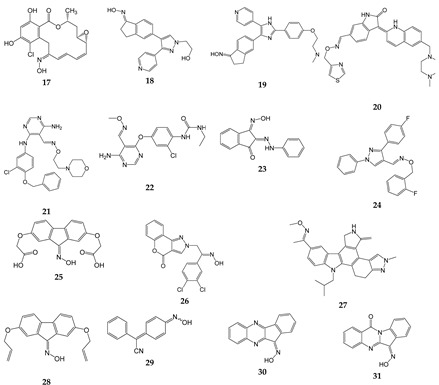
Miscellaneous oxime-based kinase inhibitors and their kinase targets.

Compound	Kinase target (IC_50_, µM)	Ref.
**17**	Src (0.056)	[34]
**18**	B-Raf (0.0001)	[62]
**19**	B-Raf (K_i_ = 0.0002), c-Raf (K_i_ = 0.0017)	[63]
**20**	VEGFR2 (0.009)	[64]
**21**	ErbB1 (0.022), ErbB2 (0.038), ErbB4 (0.021)	[65]
**22**	VEGFR-2 (0.04), Ret (0.18), Kit (0.5	[66]
**23**	EGFR (50.3% at 100 µM)	[67]
**24**	PI3Kγ (1.3)	[68]
**25**	Chk1 (13.4)	[69]
**26**	PI3Kα (0.012), PI3Kβ (0.187), PI3Kγ (0.293), PI3Kσ (0.219)	[39]
**27**	VEGF-R1 (0.008), VEGF-R3 (0.01), TIE-2 (0.03)	[70]
**28**	EGFR (55.3% at 100 µM)	[67]
**29**	CK2	[71]
**30**	JNK1/2/3	[41,72]
**31**	JNK1/2/3	[38]

**Table 3:** In vitro anticancer activity of selected oximes.

Compound	Cells	Concentration range (µM)	Effect/Mechanism ^a^	Ref.
**1**	Pancreatic ductal adenocarcinoma cells	1–10	↓ p-CDK1/cyclinB1	[80]
	MG63 and U2-OS osteosarcoma	1–10	↓ CDK2/4, FAK	[81]
	Cholangiocarcinoma linesNOZ, HuCCT1, OCUG-1, and OZ	1–60		[82]
**11, 14**	MDA-MB-231-TXSA breast cancer	10–50	↑ Caspase-3	[83]
**14**	Thyroid carcinoma	1–10	↑ Caspase-3	[84]
	Neuroblastoma SH-SY5Y	10–100		[85]
**16**	MG63 and Saos-2 osteosarcoma	1–30	↑ AMPK	[86]
	MV4-11 and FLT3/D835Y expressed MOLM14	IC_50_ = 0.001 (toward FLT3)	↓ FLT3	[61]
**17**	3Y1-B, SR-3Y1, NRK,KNRK5.2 cells	IC_50_ = 0.025 (toward v-Src)	↓ v-Src activity; ↓ Raf-1 expression	[34]
**26**	Human colorectal carcinoma HCT-116, human lung cancer A549, human liver carcinoma Huh7, human leukemia HL60	0.1–1	Inhibitor of PI3Kα, PI3Kβ, PI3Kγ and PI3Kδ	[39]

^a^ ↓ and ↑ indicate decreasing or increasing enzyme activity or protein expression after treatment with compound, respectively.

**Table 4:** In vivo anticancer activity of selected oximes.

Compound	Model	Treatment	Ref.
**1**	Pancreatic ductal adenocarcinoma cells, inoculated s.c.	10–40 mg/kg, i.p., daily for 4 days	[80]
**16**	MG63 osteosarcoma cells, inoculated s.c.	5 mg/kg, i.p. daily for 45 days	[86]
	MV-4-11 B-myelomonocytic leukemia cells, inoculated s.c.	20 mg/kg, orally, daily for 21 days	[61]
**20**	Lung cancer A549 cells, inoculated s.c.	4 mg/kg, orally, daily for 14 day	[64]
**22**	A431 epidermoid carcinoma cells, HCT116 colorectal carcinoma cells, A375 skin melanoma cells; all cells inoculated s.c.	10, 50, 100 and 200 mg/kg, intragastically, daily for 35 days,	[66]
**27**	A375 skin melanoma cells, inoculated s.c.	10 mg/kg, orally, for 22 days	[70]

s.c., subcutaneous; i.p., intraperitoneal; i.g., intragastic.

**Table 5:** In vitro anti-inflammatory activity of selected oximes.

Compound	Cell Culture	Model	Concentration Range (µM)	Effect/Mechanism ^a^	Ref.
**1**	Adipocytes	Saturated free fatty acid-induced inflammation	2–10	↑ Cell viability; ↑ mRNA for IL-4, IL-10, IL-13, TGF-β; ↓ mRNA for TNF, IL-1β, IL-6	[106]
	H9C2 rat cardiac myocyte cells	Incubation of cells with high glucose	3–30	↓ PKR protein and mRNA; ↓ JNK and NF-κB mRNA; ↓ Caspase-3 mRNA; ↓ ROS	[107]
	Cultured rat brain microglia, hippocampal slice cultures	LPS stimulation	0.5–4	↓ NF-κB activation; ↓ TNF, IL-1β, PGE_2_, ROS; ↓ Hippocampal cell death	[108]
	Mouse microglia BV-2 cells, hippocampal slice cultures	LPS stimulation	10	↓ Migration; ↓ iNOS expression; ↓ IL-6 and NO production	[30]
	Human neutrophils, monocytes, VSMCs	LTB4, CysLT and LT-enriched medium	0.3–10	↓ LT-induced VSMC migration; ↑ HO-1 induction; ↓ 5-LO in monocytes and neutrophils	[109]
	Human macrophages, primary type-I like pneumocytes	Influenza virus H5N1 infection	10	↓ IP-10, IL-1β, RANTES, IFN-β, TNF; ↑ Delay of virus replication	[110]
	SH-SY5Y cells, primary cerebellar granule neurons	H_2_O_2_-induced apoptosis	0.1–3	↑ Cell viability; ↓ p-Akt and p-GSK-3β	[111]
**11**	Human FLS	TNF stimulation	0.050	↓ mRNA for IL-1, IL-6, CCL-2, CCL-7, COX-2, MMP-9; ↓ IL-1, IL-6, CCL-2, CCL-7, COX-2, MMP-9; ↓ NF-κB, p-JNK, p-c-Jun, p-ATF-2, p-p38	[13]
	RAW264.7 macrophages	LPS stimulation	2.5–20	↓ NO, PGE_2_; ↓ iNOS mRNA, COX-2;↓ IL-1β, IL-6; ↓ p-JNK, p-IκB-α; ↑ IκB-α	[112]
	Neutrophils, RAW264.7 macrophages	LPS stimulation	5	↓ TNF; ↑ IκB-α	[113]
	Mouse mammary epithelial cells	LPS stimulation	5–50	↓ mRNA for IL-1β, IL-6, IL-10, TNF; ↓ IL-1β, IL-6, TNF; ↑ IL-10; ↓ TLR4/NF-κB and TLR4/MAPK expression and phosphorylation	[12]
**30**	PBMCs, MonoMac-6, J774.A1 cells	LPS stimulation	0.2–30	↓ IL-1α, IL-1β, IL-6, TNF, IFN-γ, GM-CSF, NO production by human and murine monocyte/macrophages.	[41]
	HUVECs		0.3–10	↓ Endothelin-1 secretion	[114]
	Macrophages, T-cells	LPS stimulation	1	↓ TNF, IL-6, IL-1β; ↓ p-JNK2, p-p38, p-IκBα, p-IKKβ; ↓ IL-6 mRNA, TNF, iNOS	[115]
**31**	Human FLS, synovial SW982 cells, HUVECs, monocytic THP-1 cells	IL-1β stimulation	1–25	↓MMP-3 gene expression; ↓ MMP-1/3 and IL-6 secretion	[116]
**32**	Human neutrophils	*f*MLF stimulation	0.03–20	↓ HNE and Pr3 activities; ↓ ROS generation, HNE release	[15]

^a^ ↓ and ↑ indicate decreasing or increasing enzyme activity or protein/mRNA expression, or functional activity after treatment with compound, respectively.

**Table 6:** In vivo anti-inflammatory activity of selected oximes.

Compound	Animal	Model	Dose	Effect/Mechanism ^a^	Ref.
**1**	Swiss albino mice	High fat-high fructose diet-induced neuropathological changes	0.4 mg/kg for 7 days	↓ Area occupied by dark neurons; ↓ Amyloid spots in hippocampus ↓ NF-κB; ↓ TNF, IL-6 ↓ Bax and caspase-3; ↑ Bcl-2	[29]
**11**	C57BL/6 mice	TPA-induced ear skin inflammation	1.5 µg/ear	↓ GSK-3β activity; ↓ IFN-γ production; ↓ Ear skin edema, epidermis hyperproliferation and dermis angiogenesis	[117]
	Rats	Intracerebral hemorrhage	10, 20, 40, 60, 80, & 100 µg/kg	↓ NF-κB, COX-2, GSK-3β phosphorylation; ↑ Brain-derived neurotrophic factor; ↓ IL-1β and IL-6, ↑ IL-10; ↓ Microglia activation and cell apoptosis	[118]
	C57BL6/J mice	Transient occlusion of the MCA	1 mg/kg i.p., 3 and 6 h after occlusion	↑ Wnt/β-catenin pathway activation ;↓ Brain edema, IgG extravasation, perivascular petechial bleeding; ↓ Hemorrhagic transformation after ischemic stroke	[119]
	C57BL/6 mice	Ligature + LPS-induced periodontitis	0.5−5 μg in 1 mL hydrogel	↓ Inflammatory cell infiltration; ↑ Expression of ALP, and Runx2	[120]
	Mice	Aging	1 mg/kg, i.p. during 2 weeks	↓ IL-6 in liver and serum; ↑ SOD and GSH in liver; ↓ Total cholesterol and triglycerides in liver & serum	[121]
	Mice	Arthritis (collagen + complete Freund’s adjuvant)	1 and 10 mg/kg	↓ Synovial hyperplasia, infiltration of inflammatory cells, cartilage destruction, and bone erosion; ↓ TNF, IL-1, IL-6, and IFN-γ in serum	[13]
**30**	Mice	Ovalbumin-specific DTH response	Every 12 h with 12.5 mg/kg, i.p., 5 injections	↓Ear thickness	[41]
	Mice	Acute lung inflammation (LPS plus D-galactosamine)	200 µg/mouse, i.p.	↓ Lethality and lung inflammation; ↓ TNF, IL-6 and IL-1β; ↓ p-JNK2, p-p38, p-IκBα & p-IKKβ; ↓ mRNA for IL-6, TNF and iNOS	[115]
	Mice	CIA	5, 20, 30 and 50 mg/kg, daily, i.p.	↓CIA and CAIA severity; ↓Cartilage erosion; ↓ Collagen II-specific antibody	[122]
	Rats	Focal cerebral ischemia/reperfusion	5 and 25 mg/kg, i.p.	↓ p-c-Jun	[123]
**31**	Mice	CIA and CAIA	30 mg/kg i.p., daily, 34 days	↓ CIA and CAIA severity; ↓ Cartilage erosion; ↓ IL-17A, GM-CSF, RANKL	[116]
**32**	Mice	HNE-induced paw edema	50–100 mg/kg, i.p.	↓ Paw edema	[15]
		LPS-induced acute lung injury	100 mg/kg, i.p.	↓ MPO; ↓ Edematous changes, alveolar thickening, leukocyte infiltration, and lung tissue destruction	

^a^ ↓ and ↑ indicate decreasing or increasing enzyme activity or protein/mRNA expression, or functional activity after treatment with compound, respectively.

**Table 7:**
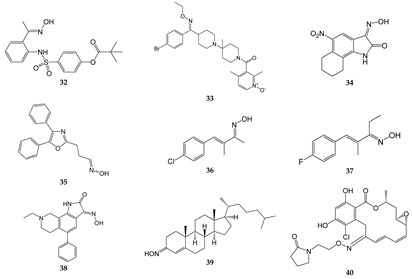
Chemical structures of oximes with non-kinase targets and mechanisms of action.

Compound	Molecular Target/Mechanism	Ref.
**32**	Dual inhibitor of HNE and Pr3	[15]
**33**	CCR5 antagonist	[169,170]
**34**	GluR6 antagonist, amelioration of inflammatory hyperalgesia	[171,172]
**35**	TRPA1 and TRPV1 antagonist	[14]
**36**	TRPA1 antagonist	[173,174]
**37**	TRPA1 antagonist	[173,174]
**38**	ASIC blocker, attenuation of pathophysiological nociceptive behaviors in CFA-inflamed and CCI rats	[175]
**39**	Binds directly to two components of the mitochondrial permeability pore, the VDAC, and translocator protein; inhibits MPTP opening	[176]
**40**	Binds to Hsp90 and provides a significant decrease in HIF-1α expression	[177]

## Data Availability

The data that support our specific findings in this review are available from the authors upon reasonable request.

## Abbreviations

Kinase Abbreviations	
AMPK	AMP-activated protein kinase
CDK1/2/5/6/9	cyclin-dependent kinases
Chk1,	checkpoint kinase 1
CK2	casein kinase 2
DRAK2	death-associated protein-related apoptotic kinase 2
DYRK	dual-specificity tyrosine-phosphorylated and regulated kinase
EGFR	epidermal growth factor receptor tyrosine kinase
ERK	extracellular signal-regulated kinase
FLT3	FMS-related receptor tyrosine kinase 3
GSK-3α/β	glycogen synthase kinase 3
IGF1R	receptor of insulin-like growth factor type 1
IRAK4	interleukin-1 receptor-associated kinase 4
JAK1/2/3	Janus kinases 1/2/3, tyrosine kinases
JNK	c-Jun N-terminal kinase
Lck	lymphocyte-specific protein tyrosine kinase
Lyn	non-receptor tyrosine-protein kinase
PhK	serine/threonine-specific phosphorylase kinase
PI3K	phosphatidylinositol 3-kinase
PKR	RNA-dependent protein kinase R
RSK2	ribosomal S6 kinase 2
SGK	serine/threonine-protein kinase Sgk1 (serum and glucocorticoid-regulated kinase 1)
VEGFR1/2	vascular endothelial growth factor receptor tyrosine kinase
Other Abbreviations	
ALP	alkaline phosphatase
AP-1	activator protein 1
ASIC	acid-sensing ion channel
ATF-2	activating transcription factor 2
CAIA	collagen-antibody-induced arthritis
CCI	chronic constriction injury
CCL	chemokine ligand
CCR5	chemokine receptor 5
CFA	complete Freund’s adjuvant
CIA	collagen-induced arthritis
COX-2	cyclooxygenase 2
CysLT	cysteinyl leukotriene
DTH	delayed-type hypersensitivity
eNOS	endothelial NO synthase
FLS	fibroblast-like synoviocytes
GluR6	glutamate receptor 6
GM-CSF	granulocyte-macrophage colony-stimulating factor
HIV	human immunodeficiency virus
HNE	human neutrophil elastase
HO-1	heme oxygenase 1
Hsp90	heat shock protein 90
HUVECs	human umbilical vein endothelial cells
IFN	interferon
IL	interleukin
iNOS	inducible nitric oxide synthase
IP-10	interferon γ-induced protein 10
LO	lipoxygenase
LPS	lipopolysaccharide
LTB4	leukotriene B4
MAPK	mitogen-activated protein kinase
MCA	middle cerebral artery
MCP	monocyte chemoattractant protein
MMECs	mouse mammary epithelial cells
MMP	matrix metalloproteinase
MPO	myeloperoxidase
NF-κB	nuclear factor κB
NO	nitric oxide
NSAIDs	nonsteroidal anti-inflammatory drugs
OCN	osteocalcin
PBMCs	peripheral blood mononuclear cells
PDE	phosphodiesterase
PGE2	prostaglandin E2
Pr3	proteinase 3
PTGS-2	prostaglandin endoperoxide synthase 2
RANKL	receptor activator of NF-κB ligand
RANTES	regulated on activation, normal T cell expressed and secreted
ROS	reactive oxygen species
Runx2	runt-related transcription factor 2
S.c.	subcutaneous
SOD	superoxide dismutase
STAT	signal transducer and activator of transcription
TGF-β	transforming growth factor β
TLR	Toll-like receptor
TNF	tumor necrosis factor
TRPA1	transient receptor potential ankyrin 1
TRPV1	transient receptor potential vanilloid 1
VDAC,	voltage-dependent anion channel
VEGFA	vascular endothelial growth factor A
VSMCs	vascular smooth muscle cells
Chemical Names	
Compound **1** (**E231**)	indirubin-3′-oxime
Compound **2**	indirubin-3′-acetoxime
Compound **3** (**E804**)	indirubin-3′-oxime 2,3-dihydroxypropyl ether
Compound **4** (**E738**)	5-methoxyindirubin-3′-oxime 1,2-dihydroxyethyl ether
Compound **5**	5′,6′-difluoro-5-methoxy-indirubin-3′-oxime 2-hydroxyethyl ether
Compound **6** (**LDD970**)	5-[(1-morpholino)carbonyl]indirubin-3′-oxime
Compound **7** (**AGM130**)	5-nitro-5′-hydroxyindirubin-3′-oxime
Compound **8**	5-(pentanamido)indirubin-3′-oxime
Compound **9** (**LDD1937**)	5-(methoxycarbonyl)indirubin-3′-oxime 2-(piperazin-1-yl)ethyl ether dihydrochloride
Compound **10**	5-iodoindirubin-3′-oxime
Compound **11**	6-bromoindirubin-3′-oxime
Compound **12**	6-bromoindirubin-3′-acetoxime
Compound **13** (**MLS-2384**)	6-bromoindirubin-3′-oxime 2-(piperazin-1-yl)ethyl ether
Compound **14**	7-bromoindirubin-3′-oxime
Compound **15**	7-bromo-5′-carboxyindirubin-3′-oxime
Compound **16**	5-fluoroindirubin-3′-oxime 2-(piperazin-1-yl)ethyl ether dihydrochloride
Compound **17**	radicicol 6-oxime
Compound **18** (**GDC 0879**)	2,3-dihydro-5-[1-(2-hydroxyethyl)-3-(4-pyridinyl)-1*H*-pyrazol-4-yl]-1*H*-inden-1-one oxime
Compound **19** (**SB 590885**)	5-[2-[4-[2-(dimethylamino)ethoxy]phenyl]-5-(4-pyridinyl)-1*H*-imidazol-4-yl]-2,3-dihydro-1*H*-inden-1-one oxime
Compound **20** (**YM**-**359445**)	(3*Z*)-3-[6-[(4-methylpiperazin-1-yl)methyl]quinolin-2(1*H*)-ylidene]-2-oxoindoline-6-carbaldehyde *O*-(1,3-thiazol-4-ylmethyl)oxime.
Compound **21** (**JNJ**-**28871063**)	5*E*-4-amino-6-(4-benzyloxy-3-chlorophenylamino)pyrimidine-5-carboxaldehyde *N*-(2-morpholin-4-ylethyl) oxime
Compound **22** (**JNJ**-**38158471**)	(*E*)-1-(4-((6-amino-5-((methoxyimino)methyl)pyrimidin-4-yl)oxy)-2-chlorophenyl)-3-ethylurea
Compound **23**	1*H*-indene-1,2,3-trione-2-(phenylhydrazone) 1-oxime
Compound **24**	(*E*)-3-(4-fluorophenyl)-1-phenyl-1*H*-pyrazole-4-carbaldehyde *O*-(2-fluorobenzyl) oxime
Compound **25**	2,2′-((9-(hydroxyimino)-9*H*-fluorene-2,7-diyl)bis(oxy))diacetic acid
Compound **26**	((*E*)-2-(2-(3,4-dichlorophenyl)-2-(hydroxyimino)ethyl)chromeno[4,3-*c*]pyrazol-4(2*H*)-one)
Compound **27**	(*E*)-1-(13-isobutyl-4-methyl-6-methylene-2,4,6,7,8,13-hexahydro-1*H*-indazolo[5,4-*a*]pyrrolo[3,4-*c*]carbazol-10-yl)ethan-1-one *O*-methyl oxime
Compound **28**	2,7-bis(allyloxy)-9*H*-fluoren-9-one oxime
Compound **29** (**4-AN**)	phenylcyanomethylenequinone oxime-4-(hydroxyimino) cyclohexa-2,5-dien-1-ylidene](phenyl)ethanenitrile
Compound **30** (**IQ-1**)	11*H*-indeno[1,2-*b*]quinoxalin-11-one oxime
Compound **31**	tryptanthrin-6-oxime
Compound **32**	(*E*)-4-(*N*-(2-(1-(hydroxyimino)ethyl)phenyl)sulfamoyl)phenyl pivalate
Compound **34** (**NS 102**)	6,7,8,9-tetrahydro-5-nitro-1*H*-benz[*g*]indole-2,3-dione 3-oxime
Compound **35** (**SZV-1287**)	3-(4,5-diphenyl-1,3-oxazol-2-yl)propanal oxime
Compound **36** (**AP 18**)	4-(4-chlorophenyl)-3-methyl-3-buten-2-one oxime
Compound **37** (**A 967079**)	(1*E*,3*E*)-1-(4-fluorophenyl)-2-methyl-1-pentene-3-one oxime
Compound **38** (**NS 383**)	8-ethyl-6,7,8,9-tetrahydro-5-phenyl-1*H*-pyrrolo[3,2-*h*]isoquinoline-2,3-dione-3-oxime
*f*MLF	formyl-l-methionyl-l-leucyl-l-phenylalanine
MPTP	1-methyl-4-phenyl-1,2,3,6-tetrahydropyridine
TPA	12-*O*-tetradecanoylphorbol-13-acetate
